# Effect of Storage Period on Dimensional Stability of Alginplus and Hydrogum 5

**Published:** 2017-01

**Authors:** Shima Aalaei, Rohollah Ganj-Khanloo, Fatemeh Gholami

**Affiliations:** 1 Assistant Professor, Department of Prosthodontics, Dental Caries Prevention Research Center, Qazvin University of Medical Sciences, Qazvin, Iran; 2 Private Practice, Zanjan, Iran

**Keywords:** Dimensional Measurement Accuracy, Dental Impression Materials, Alginic Acid

## Abstract

**Objectives:**

This study aimed to evaluate the effect of storage period on dimensional stability of Alginplus and Hydrogum 5.

**Materials and Methods:**

In this in vitro experimental study, 60 impressions were taken of an upper jaw typodont, including 10 impressions for each storage period to be tested (12 minutes, 24 and 120 hours) for each type of alginate. Then, the impressions were stored in an incubator with stable temperature and humidity, and poured using a type III dental stone. Subsequently, the mesiodistal dimension, occlusogingival height, and interarch distance were measured using a digital caliper with an accuracy of 0.01mm. The data were analyzed using ANOVA and t-test (P<0.05).

**Results:**

Alginplus and Hydrogum 5 impressions were not significantly different from the master model after 12 minutes and 24 hours in terms of dimensions (P>0.05). After 120 hours, all dimensions measured on casts were significantly different from those measured on the master model, except for the mesiodistal dimension of the Hydrogum 5 impressions.

**Conclusions:**

At a consistent temperature and humidity, the Alginplus and Hydrogum 5 impressions were dimensionally stable for at least 24 hours.

## INTRODUCTION

Manufacturers have made attempts to synthesize stable alginate products allowing delayed pouring of casts [1], which is inevitable in most clinical situations [2]. Alginate is one of the most commonly used impression materials. It was introduced to dentistry after World War II [3]. This material is simple to use, while offering patient comfort, and requires minimal equipment. Alginate is used to obtain primary and final casts in fabrication of partial dentures and in orthodontic treatments. It is also used to obtain diagnostic casts and opposing casts, in temporary restorations and for fabrication of trays for prosthetic treatments and fluoride therapy [4]. However, this impression material undergoes considerable and unacceptable dimensional changes over long periods of time. Alginate impression materials have low volume stability, since they first undergo expansion, and then shrink over time [5]. In addition, hydrocolloid impression materials develop syneresis and imbibition, which cause water depletion and dimensional changes [6,7].

Various studies have evaluated the effect of different periods of time on dimensional stability of alginate impressions and reported variable results; however, with use of conventional alginates, it has been suggested that impressions must be poured within 12 minutes [8,9]. It has also been reported that extended-pour alginate impressions can be maintained for up to five days [2] with small dimensional changes, which are acceptable. For instance, in changes equal to 0.104% [10], 74μm marginal change and 39μm occlusal change are considered acceptable [11].

Two new products namely Alginplus (Major Prodotti Dentari S.p.a., Moncalieri, Italy) and Hydrogum 5 (Zhermack S.p.a., Badia Polesine, Rovigo, Italy) have become popular in recent years. The manufacturers of these products claim that they can be preserved in appropriate conditions for as long as five days. The suggested appropriate time for pouring alginate impressions is 12 minutes [1,12]. In many clinical situations, alginate impressions are sent to the laboratory after 24 hours [2,12,13]; however, it has been claimed that this period can be extended to five days with the use of new alginate products [2]. With this in mind, the present study aimed to evaluate the dimensional stability of alginate impression materials over different lengths of time (12 minutes, 24 hours and 120 hours).

## MATERIALS AND METHODS

### Impression materials and casting:

**Fig. 1:**
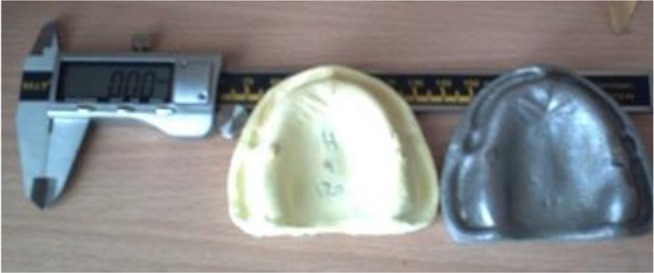
Maxillary Typodont and cast and digital caliper

In this in vitro experimental observational study, 60 casts made by pouring Alginplus and Hydrogum 5 impression materials (30 of each material) were evaluated. Each impression material was studied after 12 minutes, 24 hours and 120 hours. Impressions were poured with dental stone, and their dimensions were measured using digital calipers. A master model served as the control group. An edentulous maxillary dental model (Typodont; Nissin Dental Products Inc., Kyoto, Japan) with three cubic stainless steel indices in the left first and second premolar positions and the right first premolar position was prepared using a miller (Fig. 1). These indices served as the reference points for the measurements.

Some parallel grooves were created in the mesial surface of the first index and the distal surface of the second index on the left, and palatal surface of the first left and right indices for exact measurements. All impressions were made using 10 perforated stainless steel dental trays (Takson Co., Tehran, Iran). These trays had two metal frameworks which were fixed to the above-mentioned prepared grooves in the model. Appropriate amounts of each impression material were mixed as follows:

- Alginplus: A total of 19g of powder (Major Prodotti Dentari S.p.a., Moncalieri, Italy) was added to 40mL of pure water, with a mixing time of less than 35 seconds, and a working time of two minutes.
- Hydrogum 5: A total of 30g of powder (Zhermack S.p.a., Badia Polesine, Rovigo, Italy) was added to 50mL of pure water, with a mixing time of less than 30 seconds, and a working time of 65 seconds.

Alginate glue (Fix Adhesive Spray; Dentsply, Rome, Italy) was used, and the impression was taken one minute after it dried. After taking 10 alginate impressions, and 12 minutes of preservation, the impressions were poured. For pouring, 100g of dental stone (natural stone, yellow gypsum; Ernst Hinrichs Dental GmbH, Goslar, Germany) was mixed with 30mL of water according to the manufacturer’s instructions. A pre-vacuum Twister Evolution mixer (Renfert Co, St. Charls, Illinois, USA Renfert, USA) was used to to obtain a homogenous mixture. All impressions were made by an expert technician in a manner close to the clinical setting; then, they were wrapped in a wet towel, placed in a sealed bag, and incubated (Fan Azma Gostar Co., Tehran, Iran) at 23±1°C. Two of the other 10 impressions were also prepared as mentioned above, and placed in a similar incubator for 24 and 120 hours. For each alginate type, the impressions were separated from the casts one hour after pouring.

### Dimensional measurements:

The mesiodistal dimension, occlusogingival height and interarch distance on the master model and prepared casts were measured 24 hours after the casts were separated from the impressions. The mesiodistal size was the distance from the mesial groove of the left first premolar index to the distal groove of the second premolar index on the same side. The height of the index placed in the left first premolar site was considered as the occlusogingival height, while the interarch distance was determined by measuring the distance between the palatal groove of the indices of the first premolars on the left and right sides.

These measurements were made 10 times by two independent experts who were blinded to the alginate type, using an electronic digital caliper (Minova, Tianjin, China) with 0.01mm precision. The intra-examiner and inter-examiner agreements were calculated to be 0.46 and 0.27, respectively.

### Statistical analysis:

The normality of the data for the parameters of this study was checked using the Kolmogorov-Smirnov test. Two-way ANOVA was used to evaluate the cumulative effects of the time and alginate type on the dimensional stability of the impressions. In cases in which the test results indicated statistical significance, one-way ANOVA was used to evaluate the significance of the differences among the three study groups for each alginate type for different time periods (12 minutes, 24 hours and 120 hours). The Bonferroni test was used for pairwise comparisons of the different time periods for each alginate type.

T-test was used to compare the two alginate types and their differences with the original model for each time period. Statistical significance was set at P<0.05.

## RESULTS

Based on the results of two-way ANOVA, the effect of alginate type, storage time, and their interaction effect on each dimension were statistically significant (P<0.05). In the original model, the mesiodistal dimension was 6.55mm. One-way ANOVA showed that the mean mesiodistal dimension of impressions made with Alginplus was different in different storage times (P=0.005); however, in Hydrogum 5 impressions, the the mean of mesiodistal size was not different (P=0.26). In Alginplus impressions, the difference in mesiodistal dimensions was not significant after 12 minutes and 24 hours of storage (P=0.9). However, after 120 hours, the difference in mesiodistal dimensions was significant compared to that after other storage periods (P=0.003 for 12 minutes of storage, P=0.003 for 24 hours of storage). Tables 1 to 3 show the percentage of changes in different dimensions in each alginate type after different storage times. In the original model, the occlusogingival height was 2.63mm. Based on the results of one-way ANOVA, the occlusogingival height was different between the two alginate types after different storage times (P=0.008 for Alginplus, P=0.009 for Hydrogum 5). Based on the Bonferroni test, the differences in occlusogingival heights after 12 minutes and 24 hours of storage were not significant in the two alginate types (P=0.57 for Alginplus, P=0.53 for Hydrogum 5); but, after 120 hours, the differences with other storage times were significant in both alginate types (P=0.002 and P=0.017 for Alginplus, P=0.004 and P=0.025 for Hydrogum 5, respectively). In the main model, the interarch distance was 33.30mm. Based on the results of one-way ANOVA, the interarch distances in both alginate types were different after different storage times (P=0.013 in Alginplus, P=0.032 in Hydrogum 5). The differences in the interarch distances in the two alginates were not significant after 12 minutes and 24 hours of storage based on the Bonferroni test (P=0.9 in Alginplus, P=0.79 in Hydrogum 5); however, after 120 hours, the differences were significant with other storage times (P=0.012 and P=0.021 in Alginplus, P=0.008 and P=0.032 in Hydrogum 5, respectively).

**Table 1:** Mesiodistal measurements made on casts produced from the understudy alginates following different storage times

**Type of alginate**	**Storage time**	**Mean**	**Standard deviation**	**Dimensional changes (%)**
Alginplus	12 m	6.56	0.01826	0.16
	24 h	6.56	0.0137	0.15
	120 h	6.60	0.03348	0.77
Hydrogum 5	12 m	6.55	0.01317	0.04
	24 h	6.56	0.01776	0.10
	120 h	6.56	0.01337	0.11

**Table 2:** Occlusogingival height measurements on casts produced from understudy alginates following different storage times

**Type of alginate**	**Storage time**	**Mean**	**Standard deviation**	**Dimensional changes (%)**
Alginplus	12 m	2.64	0.03736	0.47
	24 h	2.65	0.04035	0.58
	120 h	2.69	0.02635	2.28
Hydrogum 5	12 m	2.63	0.03360	0.08
	24 h	2.64	0.03725	0.38
	120 h	2.68	0.03590	1.90

**Table 3:** Inter-arch distance measurements made on casts produced from the understudy alginates following different storage times

**Type of alginate**	**Storage time**	**Mean**	**Standard deviation**	**Dimensional changes (%)**
Alginplus	12 m	39.30	0.02558	0.00
	24 h	39.30	0.03327	0.00
	120 h	39.34	0.03779	0.12
Hydrogum 5	12 m	39.31	0.05662	0.04
	24 h	39.30	0.10619	0.00
	120 h	39.39	0.06485	0.22

After 120 hours, all three dimensions in both alginate types exhibited significant differences with the main model, except for the mesiodistal dimension in Hydrogum 5 at 120 hours (P=0.132, Table 4). Based on the results of t-test, there were no significant differences between the two alginate types in different dimensions and different storage times (Table 4), except for the mediastinal dimension at 120 hours.

**Table 4:** Comparison of the mean mesiodistal dimension, occlusogingival height, and inter-arch distance of the casts prepared from Alginplus and Hydrogum 5 impression materials after different storage times with each other and the original model

**Time (h)**	**Variables**	**Impression materials**	**Dimension**	**P-value (Alginplus, Hydrogum 5)**	**Original model size**	**P-value (Impressions, original model)**
0.2	Mesiodistal dimension	Alginplus	6.56±0.02	0.276	6.55	0.276
		Hydrogum 5	6.55±0.01			0.642
	Occlusogingival height	Alginplus	2.64±0.04	0.537	2.36	0.336
		Hydrogum 5	2.63±0.03			0.855
	Inter-arch distance	Alginplus	39.30±0.03	0.426	39.30	0.904
		Hydrogum 5	39.31±0.06			0.424
24	Mesiodistal dimension	Alginplus	6.56±0.01	0.667	6.55	0.677
		Hydrogum 5	6.56±0.02			0.117
	Occlusogingival height	Alginplus	2.65±0.04	0.734	2.36	0.270
		Hydrogum 5	2.64±0.04			0.464
	Inter-arch distance	Alginplus	39.30±0.03	0.954	39.30	0.721
		Hydrogum 5	39.30±0.11			0.977
120	Mesiodistal dimension	Alginplus	6.60±0.03	0.011	6.55	0.002
		Hydrogum 5	6.56±0.01			0.132
	Occlusogingival height	Alginplus	2.69±0.03	0.334	2.36	0.000
		Hydrogum 5	2.68±0.04			0.001
	Inter-arch distance	Alginplus	39.34±0.04	0.679	39.30	0.015
		Hydrogum 5	39.39±0.06			0.001

## DISCUSSION

Based on the results of the present study, Alginplus and Hydrogum 5 can preserve their dimensional stability for up to 24 hours, but not 120 hours. In our study, the dimensions of Hydrogum 5 were more stable than those of Alginplus. The water content of the material is a key factor in this regard [14]; the ability to preserve water probably affects the dimensional stability [4]. The water content of Hydrogum 5 is less than that of Alginplus, thus, the former is better capable to preserve water. With regard to alginate impressions, first shrinkage occurs during the setting reactions, even if the material is in contact with the saliva during this process [13,15]. Then, imbibition occurs if it is in contact with moisture, resulting in expansion of impression.

If there is contact with air, syneresis as well as evaporation occur; however, the effect of syneresis is stronger than that of evaporation [16].

Syneresis occurs toward the tray, especially if alginate tray adhesive is used, resulting in enlargement of the die in the cast [17,18]. This is probably why the height of index increased in the current study; however, in the maxilla in the palatal area, shrinkage occurs towards the bulk of the material in the palate [19,20], resulting in a smaller interarch dimension [17,18]. There was a decrease in the interarch distance of impressions, which was compensated for by the setting expansion of gypsum, to some extent [17]. It is likely that the magnitude of change is lower in this dimension than that in other two dimensions. Thus, it might have been equal to that in the original model at 12 minutes and 24 hours. The magnitude of interarch dimension change at 120 hours was lower than that in occlusogingival height and a little more than that of mesiodistal dimension (0.01%). The setting expansion of gypsum possibly explains why the distance was not negative. According to a study by Atashrazm et al, [15] reducing the expansion of plaster resulted in a negative distance, confirming this theory. Therefore, there is shrinkage towards the palate in the maxilla, which should be corrected by modifying the tray and decreasing the thickness of the impression material in this area [17]. Since the current study used a prefabricated tray, the alginate was thick on the palate, but these changes occur less frequently in studies using special trays with an adjusted distance in the palate.

It is recommended to store the impressions in an environment with 100% relative humidity in order to prevent evaporation and syneresis [2]. In many studies, storage in a wet towel has been suggested, so that imbibition would compensate for the setting expansion [21–24]. However, in this condition, it seems that shrinkage is not uniformly distributed in all areas [24]; therefore, it is suggested to store the impressions in a bag with sealing ability after expulsion of extra air [25]. It is possible to place a wet towel within the sealed bag for 10 minutes before the impression is placed in it, in order to create an environment saturated with moisture, and then remove it [26]. The dispute about how to store the impressions is one reason for the differences between the current study and others. Another factor contributing to different dimensional changes in alginate impressions in previous studies was the presence and amount of undercuts in the soft and hard tissues of the study models [23]. In a study by Wadhwa et al, [17] and Fonte-Boa et al, [27] the indices were placed at the palate of model, which caused differences between their results and ours. It has been shown that the elastic recovery of alginates is not complete, but is acceptable up to 1.8% based on the American Dental Association standards [12]. The presence of undercuts with varying sizes in the oral cavity increases the distortion of the impressions taken from teeth in the oral environment, compared to the impressions taken of the models [13,17].

The different results reported by different studies might be attributed to the use of dies or tooth indices. In a study by Mosharraf et al, [24] two different dies, with and without undercuts, were used, and the difference between the two dies regarding dimensional changes was significant. In this context, in the studies by Mehraban Jahromi et al, [12] and Rohanian et al, [14] different results were achieved due to the use of dental models with teeth and special trays. In their study, an insignificant difference was observed between the Hydrogum 5 model and the original model after 120 hours. Because, the impressions were taken from tooth areas that were different from our study, in which a cubic index was used for the impression taking and they only measured horizontal dimensions. Also different results were achieved due to different factors such as taking impression in water bath, storage the impressions in a plastic bag, keeping them in a condition close to a vacuum state, and type of plaster.

Because there are no undercuts during impression taking for a fixed prosthesis on the abutment teeth only, and since the accuracy of alginate is not suitable for recording the details and finish line for marginal adaptation in fixed prostheses [14], the results of studies in which the models are dentate can be more applicable to the clinical situations [17].

In studies by Atashrazm et al, [15] and Sedda et al, [4] Hydrogum 5 impressions preserved the dimensional stability better than the other impression materials. In Hydrogum 5 impressions, the dimensional stability was preserved at an acceptable level for up to 120 hours, which does not agree with the findings of our study. Sedda et al. [4] used special trays, employed a different method to mix the impression materials, kept the impressions in a humid plastic bag without a napkin, used a different type of plaster, and used a different plaster mixing method. In addition, the measurements were only performed in horizontal dimensions. In the study by Atashrazm et al, [15] the special tray and type of plaster were different from those of the current study, and they compensated 8% of dimensional changes by plaster expansion.

Many factors contributed to differences in the results, including the type of dental stone used [28], the jaw model of the study, the depth of the palate in the maxillary models, the distance between the tray and the model, presence of teeth and tissue undercuts, and the method used to store the impression and mixing materials.

In the study by Mosharraf et al, [24] which was consistent with the present study, the extent of the changes in the dimensional stability of Hydrogum 5 impression was not acceptable at 120 hours. In our study, Hydrogum 5 impressions had acceptable dimensional changes after 48 and 72 hours of storage. In addition, the maximum and minimum changes were observed in the height and distance between the dies, while method of impression storage and use of model were similar to those in our study.

Reddy et al. [29] studied two types of regular alginate, while considering the dimensional changes at two, 24, and 120 hours of storage. They reported that the difference between the impressions and the original model regarding the dimensional changes was insignificant after two hours and significant after 24 and 120 hours. A model with teeth was used in their study, and the impressions were disinfected using 2% glutaraldehyde. To mix the impression materials, an auto-mixer was used and the impressions were kept in sealed plastic bags; therefore, there were differences between their methodology and ours. More importantly, the alginates used in their studies were different from those in our study. In their study, similar to the current study, the dimensions increased, while maximum significant increase was observed at 120 hours.

In the study by Todd et al, [30] dimensional changes were compared among four alginate types, and the results indicated that the differences between the impressions and the original model were significant in all four types after 120 hours. Only one type of alginate (Kromopan) retained its dimensional stability after 24 hours. Inconsistent with the results of the current study, the three other alginates showed significant dimensional changes after 24 hours. Dimensional changes after 24 and 120 hours of storage were insignificant. Of course, the alginates and the method used were different from those of the current study. They used a standard die, and only the horizontal dimensions were measured. In addition, the measurements were made on impressions, not a plaster model. The special tray was made using a 1.5-inch distance, without holes, and separating material (spacer) was used on the die.

In a recent study, a digital cast was used via cone beam computed tomography of the alginate impression to evaluate dimensional changes. Lee et al. [31] evaluated the dimensional changes of alginate after 12, 24, 36, and 48 hours of storage, and reported acceptable dimensional stability after 24 and 36 hours. Jinang et al, [32] also compared the dimensional changes in regular alginates using this method, and there was a significant difference between this method and the cast preparation method. The impression was made of patient’s mouth, so there was no need to make a cast, and the measurements were made on the same impression after different time periods. However, the results of Todd et al. [30] were similar to those of the current study, and no significant dimensional changes were noted after 24 hours, while the dimensions of the cast increased and the impression shrank over time. The results of Jiang et al. [32] were completely different from those of the current study, which was predictable with regard to different methods of storage of impressions at room temperature without coverage.

It should be noted that clinical dimensional changes of 0.1–0.8mm [26,33] and 100–500μm [13,17] are acceptable for alginates, and since the changes in the alginates evaluated in the present study were in the above-mentioned ranges, these materials can be used in the clinical setting, and can be preserved for up to 120 hours. Almost all studies previously conducted have confirmed that the rate of dimensional changes depends on the time and type of material [4,12,14,15]. While manufacturers provide little information about the formulation of alginates [4], these changes cannot be evaluated based on chemical formulation [4]; however, it is noteworthy that in studies similar to the current study, the dimensional changes in the impressions were clinically acceptable after four or five days [19,34–37].

Since tissue undercuts of jaw models are usually much less than those in the oral cavity [4], and our study used parallel side dies without undercuts, impression distortion in the clinical setting is probably greater than that in our study.

In the mandible, in vitro and in vivo impressions are more different, since a distortion occurs in the impression at the time of mouth opening during impression taking, with a range of 0–0.5mm [21]. In impressions of the mandible, there is usually a decrease in the interarch distance due to the shrinkage of the impression material towards the tray [22], which increases in the clinical setting due to distortion of impression.

Our study had some limitations. First, no disinfectants were used, since disinfectants result in a decrease in dimensional changes [38]. In addition, there were no undercuts and no saliva, which are present in the clinical setting [4].

Based on the findings of a study by Kulkarmi and Thombare [13], temperature does not affect alginates up to 25°C; however, the oral cavity temperature is 37°C, and it is suggested that temperature must be taken into account in future studies. Moreover, the measuring tool was accurate up to 0.1mm, which was acceptable considering the clinically acceptable dimensional changes of 0.1–0.8mm [39].

## CONCLUSION

Based on the results of this study, the impression materials used (i.e., Alginplus and Hydrogum 5) do not significantly differ in terms of dimensional stability, and thus, they can be used whenever a long time interval between impression taking and pouring is required. However, further research is needed to study the dimensional stability of these impression materials in clinical situations.
